# Supplementary material for: Pneumococcal competence is a populational health sensor driving multilevel heterogeneity in response to antibiotics
Source: Nat Commun. 2024 Jul 10;15:5625. doi: 10.1038/s41467-024-49853-2 (PMC11237056; doi:10.1038/s41467-024-49853-2)
Supplement: Supplementary file 1 — Supplementary Information [file 41467_2024_49853_MOESM1_ESM.pdf]

Supplementary Information for:

Pneumococcal competence is a populational health sensor driving multilevel heterogeneity in response to antibiotics

Marc Prudhomme, Calum H. G. Johnston, Anne-Lise Soulet, Anne Boyeldieu, David De Lemos, Nathalie Campo, Patrice Polard.

Corresponding author: patrice.polard@univ-tlse3.fr

The pdf file includes:

Supplementary text

Supplementary Figures 1-15

Supplementary Tables 1-3

Introduction

In this section, we report evidence demonstrating that spontaneous pneumococcal competence development relies on a propagative mechanism, whatever the genotype of the cells. In all conditions tested, the two key parameters X_A and X_B, which report the development time and the rate of pneumococcal competence development during growth, respectively, fit with a propagative mechanism that we previously described [1] (Figure 1CD). In this mechanism, the X_A value is independent of the cell density, while the X_B value is linked to cell density. We previously defined this propagative mechanism by recording the luciferase activity of a transcriptional fusion between the luciferase gene with the promoter of the late competence gene *ssbB* (P_{ssbB}::*luc*) in an unencapsulated strain. Using the same approach with the capsulated D39 strain, Moreno-Gamez and colleagues reported a different mechanism of competence development akin to classical QS, where the X_A value varied in function of the cell density of the inoculum and the X_B value remained constant [2]. Here, we explain the differences observed between these two studies and confirm propagation as a general mechanism of pneumococcal competence development.

Results and discussion

Spontaneous competence development in the population proceeds by propagation, independent of polysaccharide capsule presence or growth medium.

Considering the reported differences between these two studies, we reproduced these experiments using the same methods and confirmed that pneumococcal competence development proceeds via propagation, irrespective of the presence of the polysaccharide capsule (Supplementary Figure 2). Another possible difference between the studies could come from environmental growth conditions including medium used. To remove all doubt, we tested C+Y medium kindly provided by the Veening laboratory. The experiment was repeated with both media made in Toulouse (Tlse's C+Y medium) and Lausanne (Lsne's C+Y medium). Notably, both laboratory media led to comparable propagative behaviour for competence

development (Supplementary Figure 3A), showing that medium differences did not explain the different results observed.

Confirmation of propagation as the general mechanism of pneumococcal competence development

In these experiments, we observed differences in readings during the X_A time (Supplementary Figures 2-3) which could affect detection of initial competence development, and could be attributable to differences in genotype, reporter construction and/or sensitivity of the luminometer. We investigated these hypotheses individually. First, a 5-fold decrease in basal competence level was observed in the $D39_{(Tlse)}$ lineage compared to its R800 lineage derivative, irrespective of the presence of the capsule (Supplementary Figure 2A). We attribute these difference to the genetic background, since minor differences can modify the fine-tuning of the idling ComABCDE QS system [1]. We also observed differences in basal expression levels between $D39_{(Tlse)}$ and $D39_{(Lsne)}$ (Supplementary Figure 3A), which possess P_{ssbB}::*luc* reporter fusions located at native or ectopic loci respectively. Exploration of this demonstrated that the ectopic P_{ssbB}::*luc* reporter was less sensitive than the native one, leading to an approximate 50-fold loss of sensitivity compared to R800 (Supplementary Figure 3A). This renders the reported values prior to competence induction too close to the limits of detection for cell densities where they are readily detectable for the P_{ssbB}::*luc* at *ssbB* reporter (Supplementary Figure 3B, red circles). In addition, by comparing the sensitivity of the luminometers used in both studies to a reference luminometer (Anthos Lucyl) [3], we revealed a 16-fold deficit in sensitivity for the Tecan luminometer used by Moreno-Gamez and colleagues (Supplementary Figure 4). To conclude, precise and exhaustive dissection of the tools used to report competence has explained the differences observed between the two studies [1,2]. In addition, Moreno-Gamez and colleagues reported transmission of CSP between two separate cell islets on an agarose pad, one of which was unable to produce CSP [2]. This indicated competence activation of cell islets receiving exogenous CSP from producing cells without direct contact, but by diffusion. However, the timing of this CSP transmission show a

delay greater than a competence cycle (30-100 min) between induction of the CSP producing cells and the non-producers. These experiments show that after a first competence wave, some CSP can be released and reach the receiver cell islet in agar pads. Our planktonic experiments are not in contradiction with this observation since we focus exclusively on the very first wave of competence induction, which we showed previously to rely on cell-to-cell contact [1]. Altogether, these results show that competence development does not fit a classical QS model, but still support a model of propagation among the population as the general mechanism of competence development in pneumococci, as previously proposed [1].

Dilution of competent cells does not abolish competence.

A previous study suggested that dilution of competent cells rendered these cells non-competent [2]. We hypothesised that if competent cells were diluted, the competence status should not be modified. To explore this, we repeated the experiments conducted in the study [2], and included a transformation assay as a second independent method to report competence. Whatever the competence state reported by P_{ssb}::*luc* RLU, if a cell fraction is competent, this should be detected in a transformation assay [3]. We focussed our analysis on the first minutes of the culture. As expected, the first recorded RLU values diminished by tenfold between each assay, which correlates to the reduction of the inoculum sizes, except for the two lowest inocula (Supplementary Figure 5A, red circles) explained by the sensitivity limit of the luminometer (Supplementary Figure 4). The specific RLU OD^{-1} activity calculated on the first recorded data confirms that dilution until 10^{-4} OD gives equivalent values for the cell population in competence recording (Supplementary Figure 5B, blue numbers, left graph). In parallel, the transformation assay gives a transformant ratio approximately fitting the theoretical transformation efficiencies estimated by the serial dilutions (Supplementary Figure 5B, right panel, red dotted line). Thus, dilution of a competent population does not render cells non-competent. In addition, we repeated the mirror experiments but using cells precultured in acidic medium (non-permissive) (Supplementary Figure 5C). We can approximately plot the competence threshold rule (200 RLU) from the previous study [2] transposed to the RLU

produced by our luminometer (Supplementary Figure 5C middle panel, red dotted line). By doing so we are able to show that the conditions previously used [2] prevented visualisation of an unmodified X_A whatever the inoculation density but a X_B linked to the cell density (Supplementary Figure 5C).

Competence propagation visualised by fluorescence microscopy

To observe competence propagation using microscopy (Figure 1E), an *endA* mutant, in which fluorescent Cy3-tDNA accumulates at the septum of competent cells, was used [4]. High- and low-density cultures of *endA* mutant cells possessing P_{ssbB}::*luc* were grown in permissive medium for competence to calculate the X_A time in these conditions (Supplementary Figure 6A). Then, samples were taken every 10 min after this time, and Cy3-tDNA added, before visualisation on an agar pad (Figure 1F and Supplementary Figure 6B). A control sample of low cell density inoculum with CSP added at the X_A time was included. Results show an exponential increase in the frequency of cells binding Cy3-tDNA (Figure 1F), from which the competence development rate can be calculated. The rate observed was significantly slower in the low-density inoculum. These results reveal propagation of competence with a rate corresponding to an exponential function that depends on cell density [1].

Sensitive transformation assays validate propagation which may originate from self-inducing competent cells, establishing bimodal behaviour.

We used transformation assays to further demonstrate propagation. We compared transformation levels in two populations with high or low cell density to a control of low cell density where exogenous CSP was added to artificially synchronise competence development. Samples were taken every 10 min to measure growth (OD_{492}), competence development (P_{ssbB}::*luc*) and the ability to transform during a 10 min or 20 min time window, with DNase I used to prevent subsequent DNA internalisation and resulting transformation

after these incubation times (Supplementary Figure 7A). In the artificially synchronised control, CSP was added at the X_A time (70 minutes), identified using P_{ssbB}::*luc* (Supplementary Figure 7B). This control leads to an immediate coordination of competence, with a 5-log jump in transformants, mimicking classical QS (Supplementary Figure 7C). The percentage of transformants was compared for each condition at each time point throughout growth, showing bimodality of the population and correlation of the exponential rate of competence development with cell density (Supplementary Figure 7C). To further highlight the exponential propagation of competence development, we calculated the difference in transformants obtained at each time point between 20 min and 10 min incubation with tDNA (Δtransformants$_{(20\text{-}10)}$) [5], since any increase in transformants 20 min after CSP addition compared to 10 min after proves the propagation of competence through a population and disproves a synchronous induction. A sequence of exponentially increasing Δtransformants$_{(20\text{-}10)}$ values was observed in both high and low density inocula, with a rate higher in the high density inocula (Supplementary Figure 7D). In addition, these transformation assays reveal transformant cells prior to competence propagation, which may represent self-induction of a sub-population of cells, highlighting a bimodal mode of development.

Mitomycin C creates a self-induced competent cell fraction

The competence stress induction experiment was repeated with MMC rather than streptomycin (Figure 2). It is of note that induction of competence by MMC at 60 ng mL^{-1} leads to a loss of correlation between cfu and OD measurement (Supplementary Figure 8A, right panels). MMC, via its crosslinking activity, affects chromosome organisation and modifies cell shape [6]. Observation by microscopy 90 min after antibiotic addition showed that contrary to streptomycin, MMC promotes chaining of 70% of the cells and kills nearly 30% of cells (Supplementary Figure 8BCD). In these conditions, MMC leads to the disappearance of the self-induced cell fraction that is observed between 80 and 160 min without antibiotic addition (Supplementary Figure 9B). On the other hand, our results suggest that MMC-mediated stress

generates self-inducing cells in the surviving population, leading to competence propagation even if some self-induced cells may not survive.

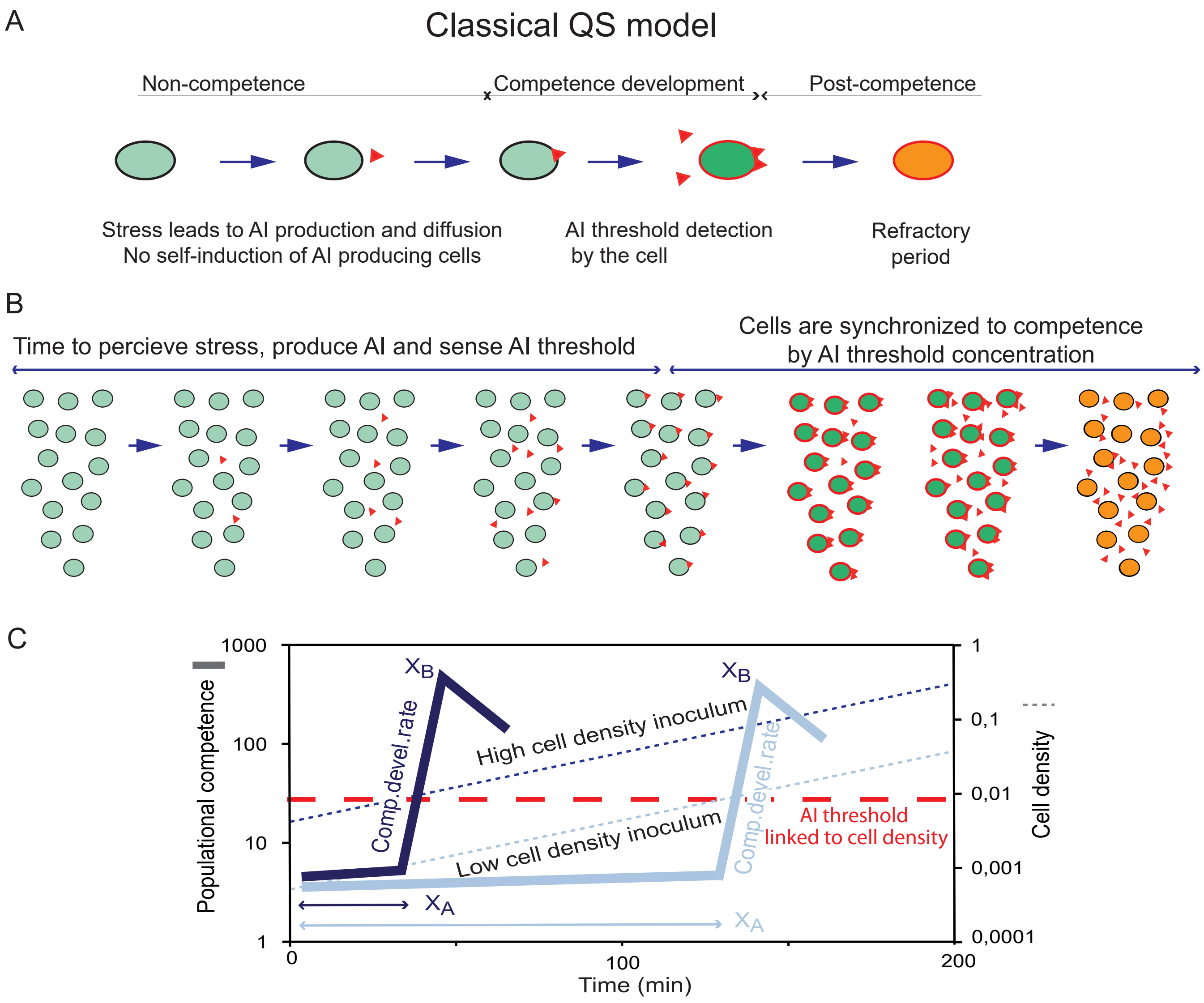
A
Classical QS model
Non-competence
Competence development
Post-competence
Stress leads to AI production and diffusion
No self-induction of AI producing cells
AI threshold detection
by the cell
Refractory
period
B
Time to percieve stress, produce AI and sense AI threshold
Cells are synchronized to competence
by AI threshold concentration
C
Populational competence
1000
100
10
1
Comp.devel.rate
High cell density inoculum
Low cell density inoculum
AI threshold
linked to cell density
Cell density
1
0,1
0,01
0,001
0,0001
0
100
200
Time (min)

Supplementary Figure 1: Schematic and model of classical QS development. (A) Competence development at the individual cell level. A non-competent cell (black contour, light green fill) senses stress and produces AI which diffuses in the medium. Cells do not self-induce competence. AI concentration increases over time, and once a threshold is reached, the cell develops competence (red contour, dark green fill). After ~30 min, the cell exits competence and enters a post-competence period (red contour, orange fill), during which it is unable to respond to a competence signal (refractory period), before becoming once again able to respond to competence signals. (B) Model of populational competence development by classical QS. In a growing planktonic cell culture in permissive medium, stress and metabolic heterogeneity means some cells produce AI, which is released into the medium. Once a threshold concentration of AI is reached, the population senses this and enters competence synchronously. After competence, cells enter a post-competence period where they are non-responsive to CSP. Cell identities as in panel A. (C) Model of classical QS as it would be reported by luciferase transcriptional fusion. A time (X_A) is required to reach a threshold concentration of AI in the medium. X_A is dependent on cell density, with a longer X_A time in lower density populations. When this AI threshold is reached, cells would develop competence synchronously, with an identical competence development rate (X_B) observed in both high and low density cultures. Dotted lines, cell growth; full lines, competence development.

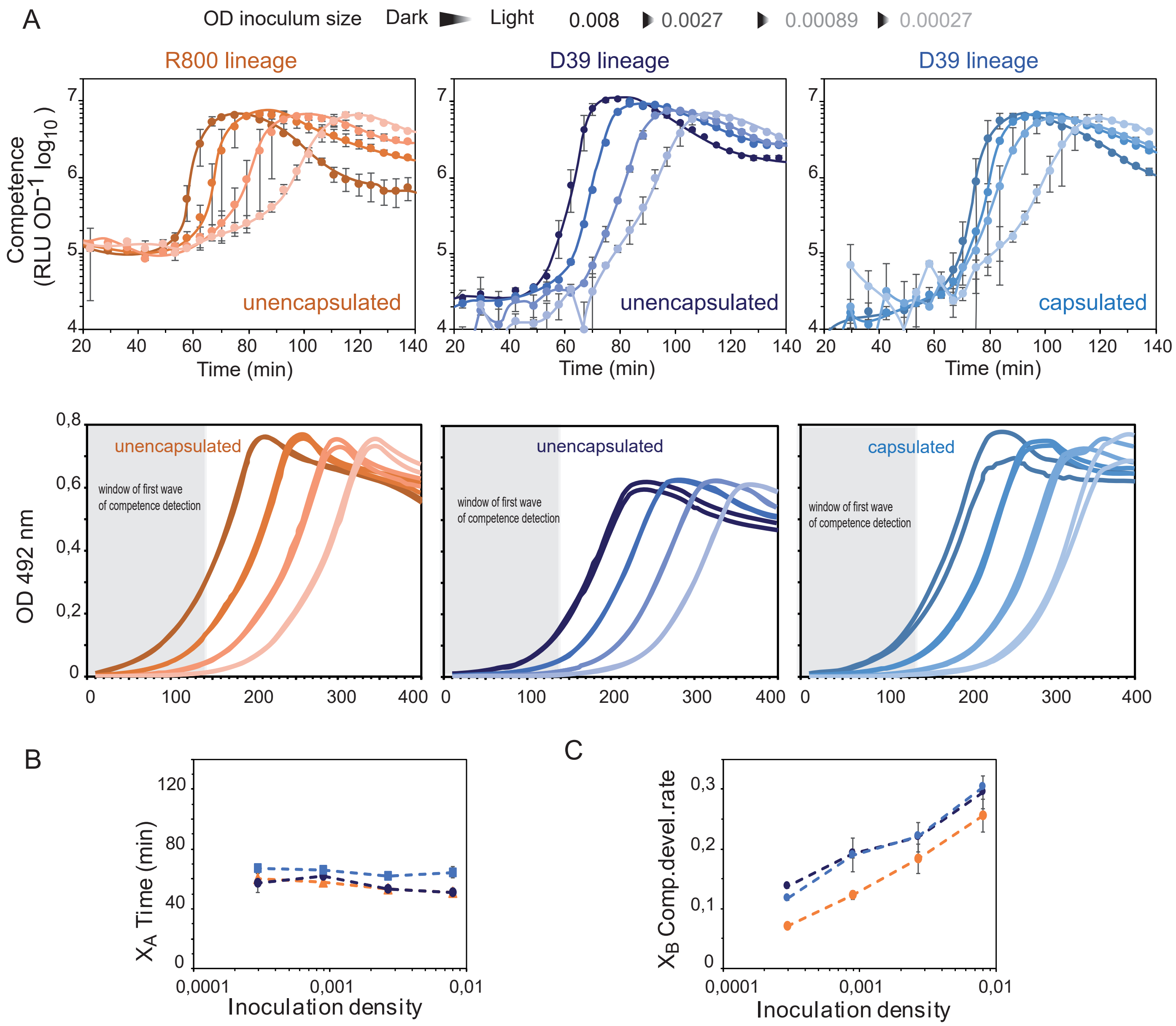
A
OD inoculum size Dark Light 0.008 0.0027 0.00089 0.00027
R800 lineage
D39 lineage
D39 lineage
Competence (RLU OD-1 log10)
unencapsulated
unencapsulated
capsulated
Time (min)
OD 492 nm
window of first wave of competence detection
B
XA Time (min)
Inoculation density
C
XB Comp.devel.rate
Inoculation density

Supplementary Figure 2: Competence proceeds by propagation irrespective of capsule presence. (A) The P_{ssbB}::*luc* fusion was used to monitor competence development throughout growth in the unencapsulated R800 lineage (orange) and capsulated and unencapsulated variants of the virulent D39 lineage (light blue and dark blue respectively). Pre-culture stocks at OD_{550} 0,4 were inoculated at 50-, 150-, 450- and 1350-fold dilutions giving inocula ranging from OD_{550} 0,008 (dark) to 0,00027 (light). Specific RLU OD^{-1} readings are reported for each strain at each inoculum. (B) Mean X_A times for different densities of inoculum of strains remain constant. (C) Mean competence development rate (X_B) for different densities of inoculum of strains correlates to cell density. Standard deviation calculated from triplicate repeats.

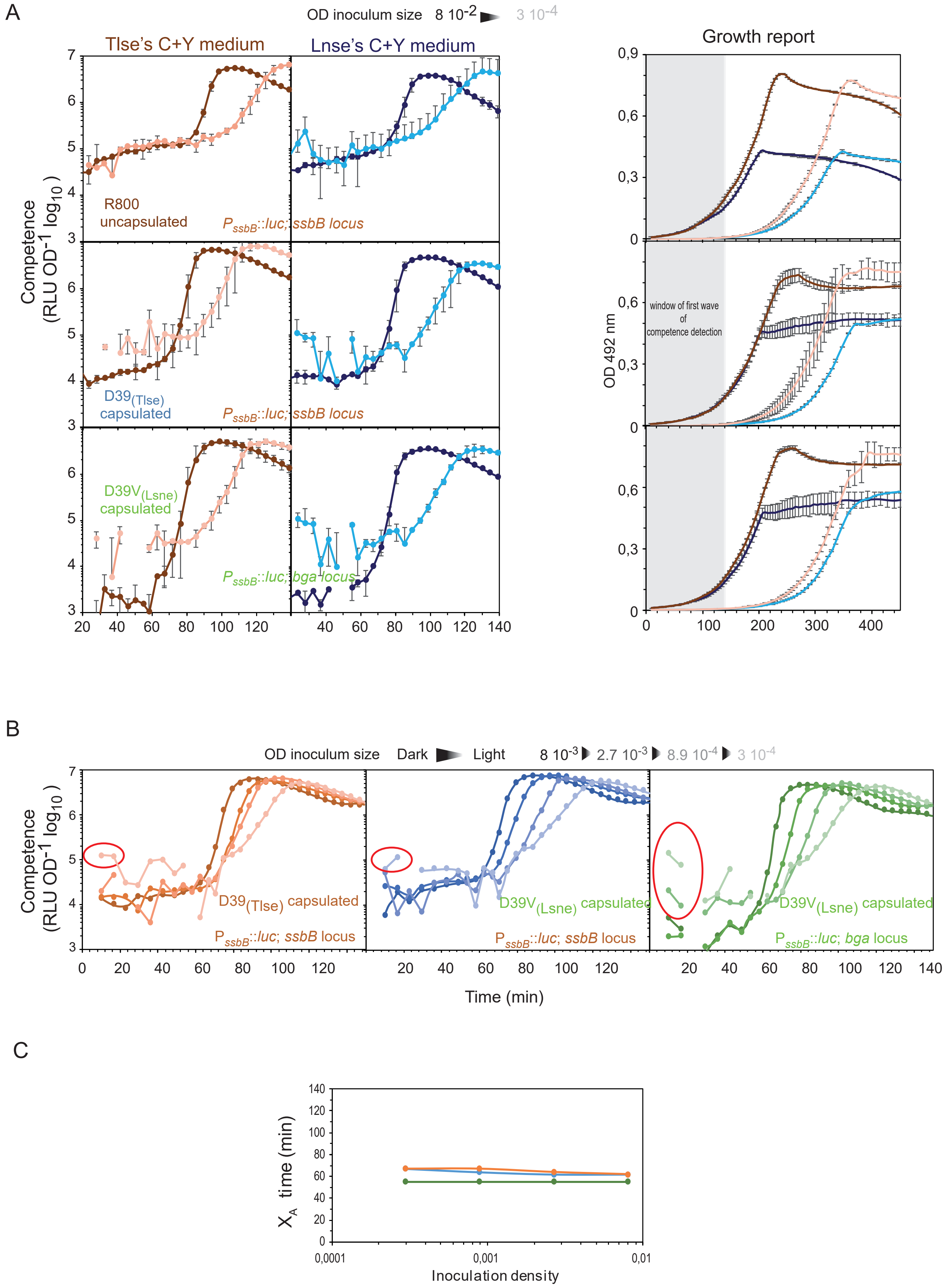
A
OD inoculum size 8 10-2 3 10-4
Tlse's C+Y medium
Lnse's C+Y medium
Growth report
Competence (RLU OD-1 log10)
R800 uncapsulated
PssbB::luc; ssbB locus
D39(Tlse) capsulated
PssbB::luc; ssbB locus
D39V(Lsne) capsulated
PssbB::luc; bga locus
window of first wave of competence detection
OD 492 nm
B
OD inoculum size Dark Light 8 10-3 2.7 10-3 8.9 10-4 3 10-4
Competence (RLU OD-1 log10)
D39(Tlse) capsulated
PssbB::luc; ssbB locus
D39V(Lsne) capsulated
PssbB::luc; ssbB locus
D39V(Lsne) capsulated
PssbB::luc; bga locus
Time (min)
C
XA time (min)
Inoculation density

Supplementary Figure 3: D39 strains or medium used do not alter the propagative behaviour of pneumococcal competence. (A) Comparison of competence development (RLU OD^{-1}) of three strains in different C+Y medium prepared in Toulouse (Tlse C+Y medium) (brown scaled colours) and Lausanne (Lsne C+Y medium) (blue scaled colours). Strains used: two from the Toulouse collection (Tlse), the unencapsulated R800 lineage (R895, brown colour) and D39 (TD277, blue colour) both with P_{ssbB}::*luc* at the *ssbB* locus but also *ssbB*$^{+}$ at the *ssbB* locus, and the third one from Lausanne (Lsne), D39V with P_{ssbB}::*luc* in the *bgaA* locus, Δ*bgaA* (DLA3) (green colour). Left panels show competence development in specific activity (RLU OD^{-1}) and right panels show growth (OD_{492}). Standard deviations are indicated for all experiments based on triplicate repeats. (B) P_{ssbB}::*luc* in the *bgaA* locus is a poor reporter of spontaneous competence propagation compared to P_{ssbB}::*luc* at the *ssbB* locus. $D39_{(Tlse)}$ containing P_{ssbB}::*luc* at the *ssbB* locus is compared to $D39V_{(Lsne)}$ containing P_{ssbB}::*luc* at the *ssbB* or *bgaA* loci. Red circles point out data that are out of range of the sensitivity of the Thermofisher Varioskan Flash luminometer (see also Figure S4). (C) Deduced X_A time of each strain for each inoculum size in panel B. Individual data shown representative of triplicate repeats showing similar results.

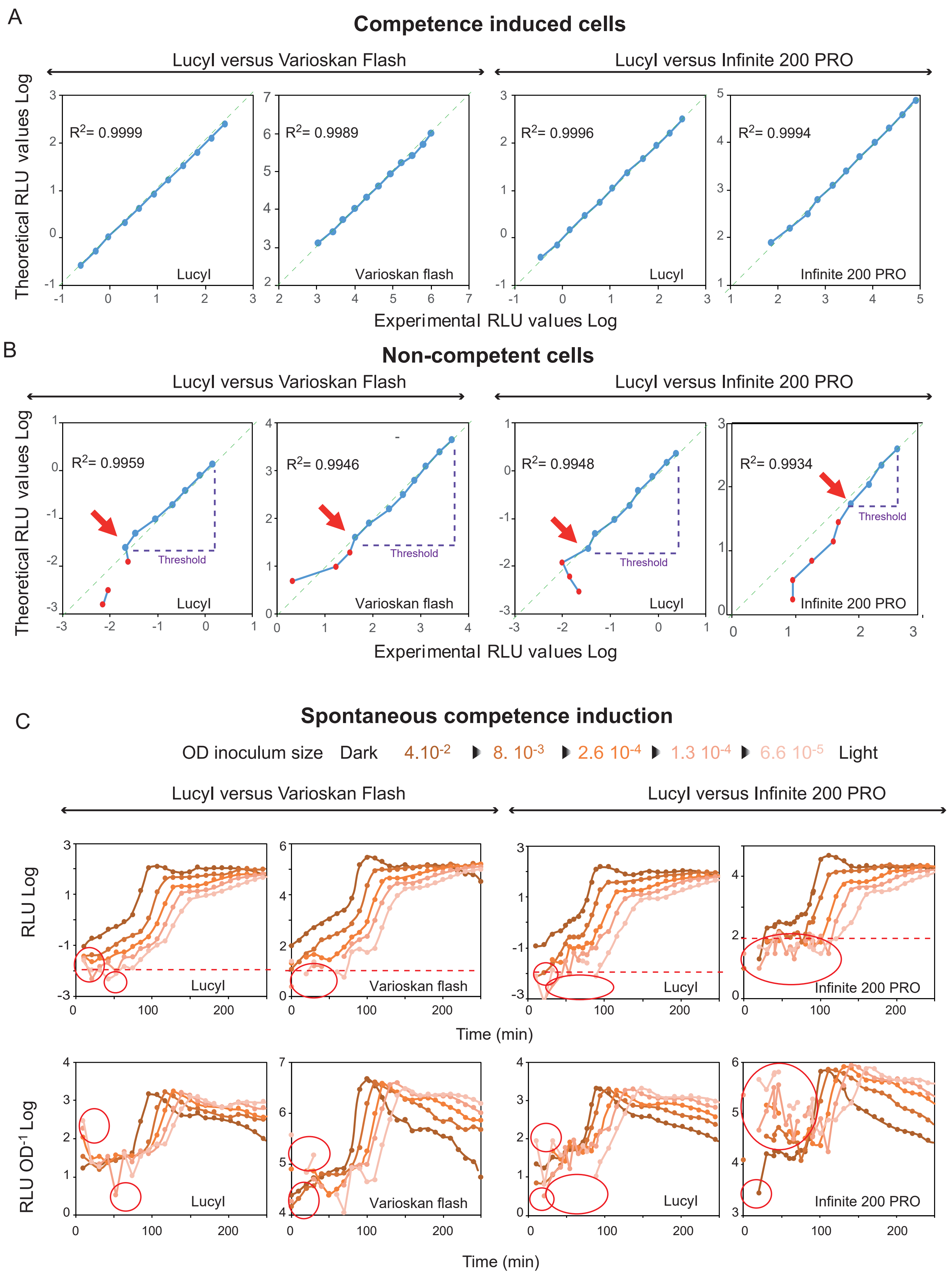

A
Competence induced cells
Lucyl versus Varioskan Flash
Lucyl versus Infinite 200 PRO
Theoretical RLU values Log
R2= 0.9999
R2= 0.9989
R2= 0.9996
R2= 0.9994
Lucyl
Varioskan flash
Lucyl
Infinite 200 PRO
Experimental RLU values Log
B
Non-competent cells
Lucyl versus Varioskan Flash
Lucyl versus Infinite 200 PRO
Theoretical RLU values Log
R2= 0.9959
R2= 0.9946
R2= 0.9948
R2= 0.9934
Threshold
Lucyl
Varioskan flash
Lucyl
Infinite 200 PRO
Experimental RLU values Log
C
Spontaneous competence induction
OD inoculum size Dark 4.10-2 8. 10-3 2.6 10-4 1.3 10-4 6.6 10-5 Light
Lucyl versus Varioskan Flash
Lucyl versus Infinite 200 PRO
RLU Log
Lucyl
Varioskan flash
Lucyl
Infinite 200 PRO
Time (min)
RLU OD-1 Log
Lucyl
Varioskan flash
Lucyl
Infinite 200 PRO
Time (min)

Supplementary Figure 4: Comparing the RLU sensitivity threshold of luminometers. Comparing sensitivity of luminometers detecting competent (A) and non-competent cells (B) in serially diluted samples. The luminometers used in each study (Thermo Varioskan Flash [1], Tecan Infinite 200 PRO [2]) were individually compared to a reference luminometer (LucyI, Anthos). The graphs represent the experimental RLU value plotted (x axis) against the theoretical calculated value (y axis) obtained by dividing the highest measured value by two-fold successively. The RLU background is obtained by measurement of an equivalent volume of sterile medium. The green dotted diagonal allows visualisation of whether the luminometer is accurate or not. Red dots represent values considered out of range. The threshold limit of each luminometer is indicated by the dotted purple lines. (C) Comparison between luminometers to detect the first wave of spontaneous competence development. The data out of range of the instrument are circled in red. The dotted horizontal red line represents the approximative threshold limit of the luminometer. We were unable to calculate the X_A for almost all cell densities with the Tecan Infinite 200 PRO luminometer.

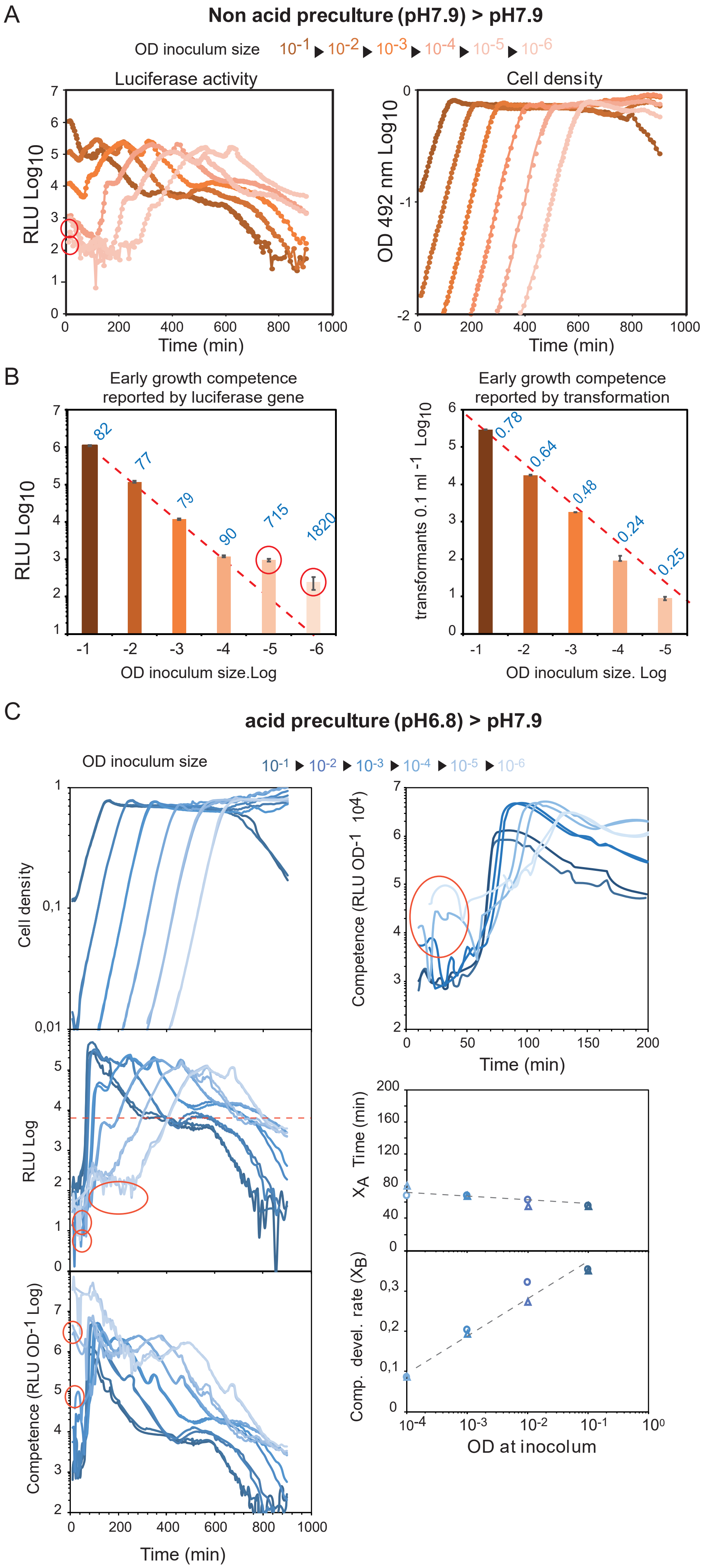
A
Non acid preculture (pH7.9) > pH7.9
OD inoculum size 10-1 10-2 10-3 10-4 10-5 10-6
Luciferase activity
RLU Log10
Time (min)
Cell density
OD 492 nm Log10
Time (min)
B
Early growth competence reported by luciferase gene
RLU Log10
82
77
79
90
715
1820
OD inoculum size.Log
Early growth competence reported by transformation
transformants 0.1 ml -1 Log10
0.78
0.64
0.48
0.24
0.25
OD inoculum size. Log
C
acid preculture (pH6.8) > pH7.9
OD inoculum size
10-1 10-2 10-3 10-4 10-5 10-6
Cell density
Competence (RLU OD-1 10^4)
Time (min)
RLU Log
XA Time (min)
Comp. devel. rate (XB)
OD at inocolum
Competence (RLU OD-1 Log)
Time (min)

Supplementary Figure 5: Dilution of competent cells does not abolish competence. We repeated the experiment described in Figure 2B of the study which suggested that dilution of competent cells abrogated competence [2]. (A) RLU (left) and OD_{492} (right). Red circles, data out of range of the instrument at the beginning of the experiment. (B) Left graph, mean RLU between 10 and 14 minutes after inoculation. The blue number on each column corresponds to calculated RLU OD^{-1} that is independent of the cell dilution. Right graph, total numbers of streptomycin resistant transformants in cfu per 0.1 mL for each inoculum size at 10 min of the growth culture (see colour guide in panel A*).* The blue number on each column reports the percent of transformants for each inoculum size. Red dotted lines represent the theoretical expected value deduced from the highest inoculum size if dilution does not affect competence. Standard deviations are represented. (C) We repeated the experiment described in Figure 2A of the same study [2]. DLA3 displays propagative competence induction. Left graphs: upper panel, cell density growth curves (OD_{492}); middle panel, RLU; bottom panel, specific activity (red circles show data out of range of the instrument). The horizontal dotted red line in the middle panel corresponds approximately to the RLU competence threshold previously described [2]. Right graphs: top panel, zoom of first 200 min of bottom left graph (red circle shows data out of instrument range); bottom panel, deduced X_A time and the deduced competence development rate (X_B) of each strain for each inoculum size. Triangles and circles represent two different assays per inoculum size. The dotted grey line corresponds to the tendency between the assays. The correlation coefficient R^2 for each exponential regression calculation (X_B) was done on a minimum of five consecutive values and found to be between 0.91 and 0.99. All the experiments described above were reproduced with the TD288 strain and gave similar results.

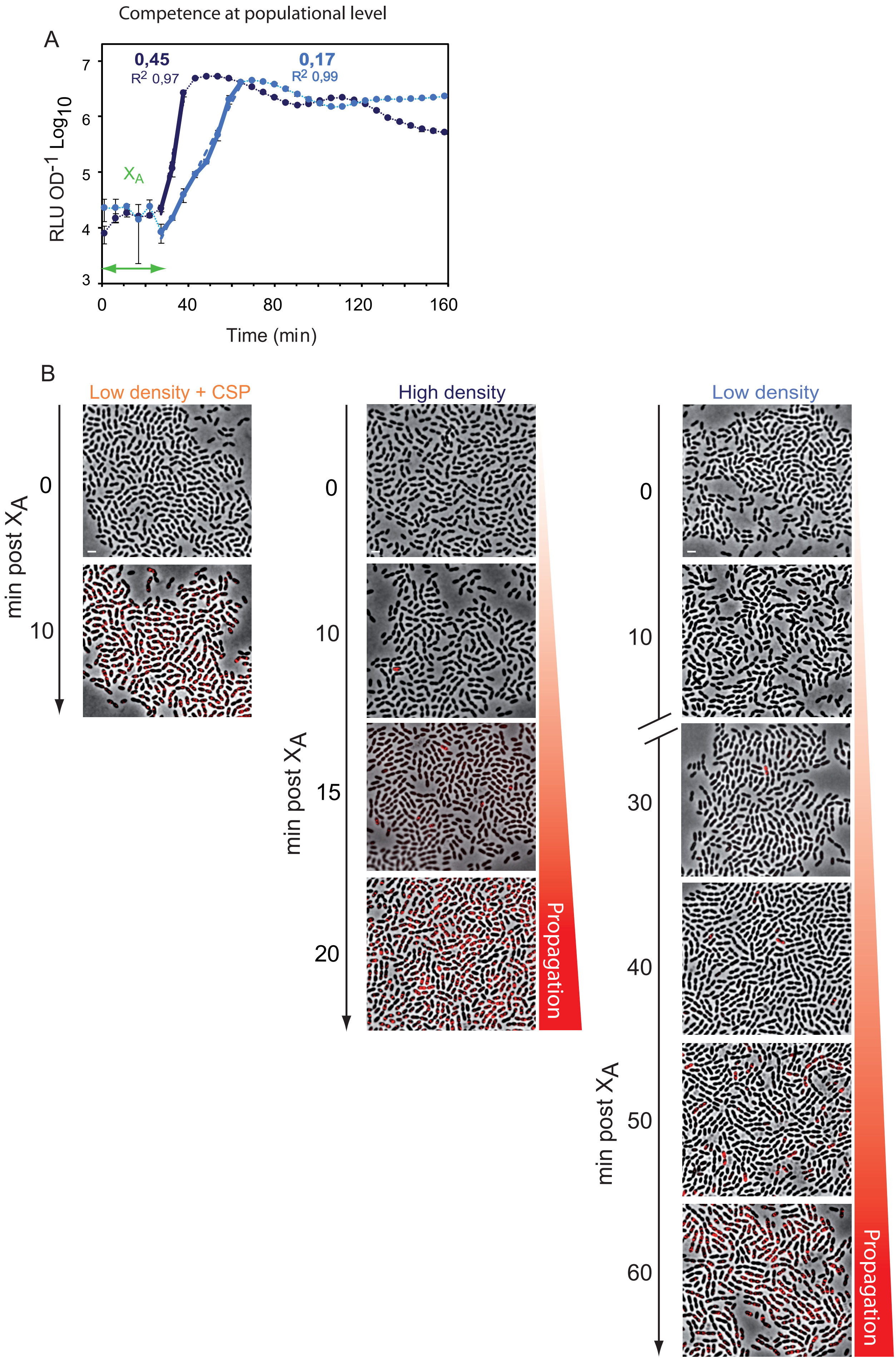

Competence at populational level
A
0,45
R2 0,97
0,17
R2 0,99
XA
RLU OD-1 Log10
7
6
5
4
3
0
40
80
120
160
Time (min)
B
Low density + CSP
High density
Low density
min post XA
0
10
15
20
30
40
50
60
Propagation
Propagation

Supplementary Figure 6: Competence propagation tracked by microscopy. Tracking binding of fluorescent tDNA to competent cells in different densities of inocula supports the propagation model. (A) Calculation of X_A time by tracking P_{ssbB}::*luc*, to determine when to begin observation of spontaneous competence development by microscopy. The graph represents the specific activity (RLU OD^{-1}) readings every 10 minutes. The X_A time (double-headed green arrow) is reported. The competence development rate values (X_B) are calculated with data restricted to the thick lines with the R^2 correlation coefficient. X_A time determined as 30 min in these conditions. (B) Fluorescent images representative of cells incubated with 285-bp Cy3-DNA during the time course as in Figure 1E, white scale bar 2 µm. Individual data shown representative of triplicate repeats showing similar results.

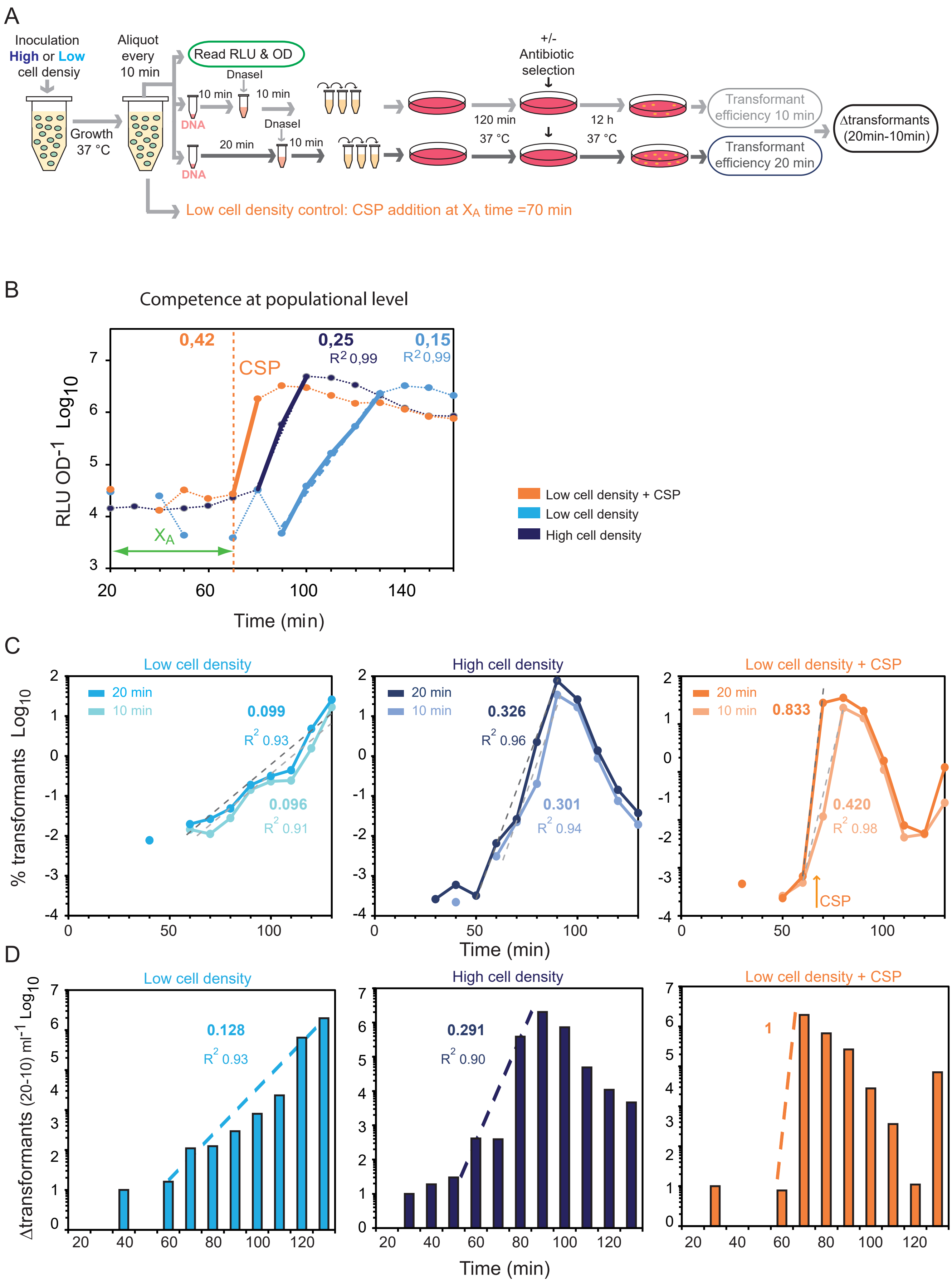
A
Inoculation
High or Low
cell densiy
Growth
37 °C
Aliquot
every
10 min
Read RLU & OD
DnaseI
10 min
10 min
DNA
20 min
DnaseI
10 min
+/-
Antibiotic
selection
120 min
37 °C
12 h
37 °C
Transformant
efficiency 10 min
Transformant
efficiency 20 min
Δtransformants
(20min-10min)
Low cell density control: CSP addition at XA time =70 min
B
Competence at populational level
0,42
CSP
0,25
R2 0,99
0,15
R2 0,99
RLU OD-1 Log10
XA
Time (min)
Low cell density + CSP
Low cell density
High cell density
C
Low cell density
20 min
10 min
0.099
R2 0.93
0.096
R2 0.91
High cell density
0.326
R2 0.96
0.301
R2 0.94
Low cell density + CSP
0.833
0.420
R2 0.98
CSP
% transformants Log10
Time (min)
D
Low cell density
0.128
R2 0.93
High cell density
0.291
R2 0.90
Low cell density + CSP
1
Δtransformants (20-10) ml-1 Log10
Time (min)

Supplementary Figure 7: Self-Induction and propagation (SI&P) observed at single cell level. (A) Schematic of transformation assays to follow competence development. The difference in transformants between 20 min exposure and 10 min exposure to transforming DNA (Δtransformants$_{(20\ min-10\ min)}$) reports the amount of cells shifting to competence between 10 min and 20 min after sampling. (B) Competence propagation observed via P_{ssbB}::*luc* as control in parallel with transformation assay using TD277. This strain was pre-cultured in non-permissive medium and was inoculated as low cell density (2.9 10^{-4} OD, light blue curves) or high cell density (8 10^{-3} OD, dark blue curves) inoculum size in permissive medium. The competence development rates with associated X_B values calculated from the exponentials (thick curves) are given with the R^2 correlation coefficient. (C) Transformation efficiency of TD277 reported during growth of different starting cell densities of cells in permissive medium. Rate of transformant increase with correlation coefficient (R^2) is reported for each condition. Note that the first transformants observed prior to propagation may represent self-induced competent cells. Colour scheme as in panel B. (D) Comparing difference between transformant levels at 10 and 20 minutes post-CSP at different cell densities demonstrates exponential propagation as the mode of competence development. The Δtransformants$_{(20\ min-10\ min)}$ is reported for each sampling for each assay. The rate of increase of Δtransformants$_{(20\ min-10\ min)}$ is reported with the R^2. Dashed lines show data range used for rate calculations. Colour scheme as in panel B. Data shown representative of two individual repeats.

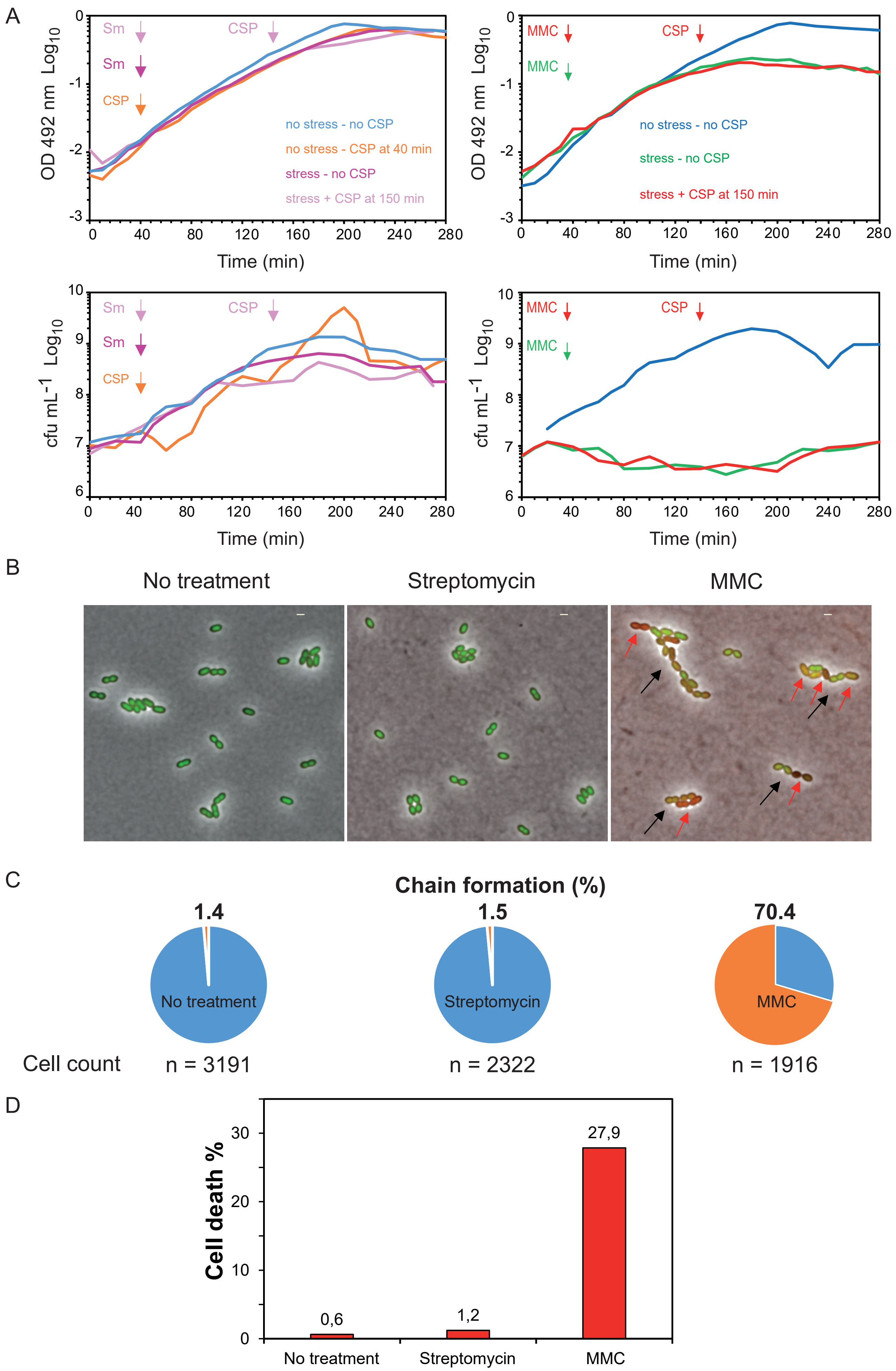

A
Sm
CSP
Sm
CSP
OD 492 nm Log10
no stress - no CSP
no stress - CSP at 40 min
stress - no CSP
stress + CSP at 150 min
Time (min)
MMC
CSP
MMC
no stress - no CSP
stress - no CSP
stress + CSP at 150 min
cfu mL-1 Log10
B
No treatment
Streptomycin
MMC
C
Chain formation (%)
1.4
1.5
70.4
No treatment
Streptomycin
MMC
Cell count
n = 3191
n = 2322
n = 1916
D
Cell death %
0,6
1,2
27,9
No treatment
Streptomycin
MMC

Supplementary Figure 8: Mitomycin C induces cell chaining and cell death. (A) Growth and viability measurements in experiments of competence induction by antibiotics. Top panels, OD followed during experiments. Bottom panels, cfu mL^{-1} obtained during the experiments presented in Figure 2 with streptomycin in Supplementary Figure 9 with MMC. (B) Visualisation of cell chaining and survival by microscopy using Live/Dead assay. Black arrows, cell chaining; red arrows, cell death. (C) Percentage of cell chaining detected for each condition. (D) Percentage of cell death observed 90 minutes after time of antibiotic addition.

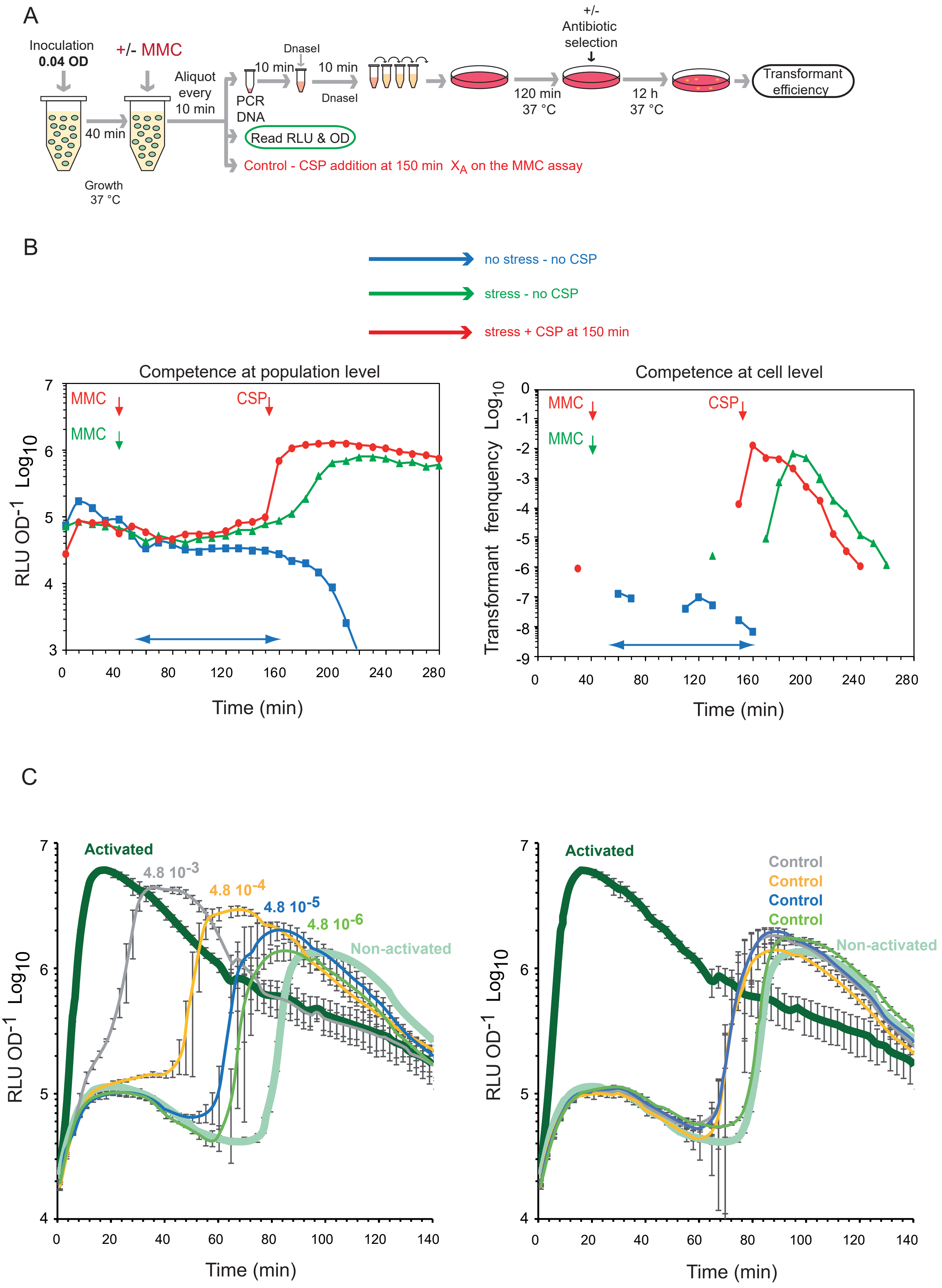
A
Inoculation
0.04 OD
+/- MMC
Aliquot
every
10 min
40 min
Growth
37 °C
PCR
DNA
10 min
DnaseI
10 min
DnaseI
Read RLU & OD
Control - CSP addition at 150 min X_A on the MMC assay
+/-
Antibiotic
selection
120 min
37 °C
12 h
37 °C
Transformant
efficiency
B
no stress - no CSP
stress - no CSP
stress + CSP at 150 min
Competence at population level
MMC
CSP
MMC
RLU OD^{-1} Log$_{10}$
Time (min)
Competence at cell level
Transformant frenquency Log$_{10}$
Time (min)
C
Activated
4.8 10^{-3}
4.8 10^{-4}
4.8 10^{-5}
4.8 10^{-6}
Non-activated
RLU OD^{-1} Log$_{10}$
Time (min)
Activated
Control
Control
Control
Control
Non-activated
RLU OD^{-1} Log$_{10}$
Time (min)

Supplementary Figure 9: Competence propagation induced by MMC. (A) Experiment conducted as described in Figure 2, but with MMC replacing streptomycin. Schematic representation of experiment carried out to explore whether competence induction by exposure to sub-lethal concentrations of MMC follows a SI&P mode of transmission. (B) MMC at 60 ng mL^{-1} was added after 40 minutes of growth (red and green curves). CSP was added after MMC at 150 minutes (red curves) of growth at the concentration of 100 ng mL^{-1}. The blue curve corresponds to the control without any addition. The left graph reports the populational competence tracked by RLU OD^{-1} and the right graph reports individual kanamycin resistant transformant tracking. Arrows represent time of addition of CSP or antibiotic. Individual data shown representative of triplicate repeats showing similar results. The blue arrowed lines highlight a period with detection of low transformant levels (right graph) revealing a self-induced cell fraction, without detection of populational competence propagation (left graph). (C) Raw data used to calculate X_A times for different dilutions in Figure 2D. Different colours represent different ratios of activated and non-activated cells. Data shown as mean of triplicate repeats with error bars representing standard deviation. Left graph, propagation test samples; right graph, control samples, as denoted in Figure 2C.

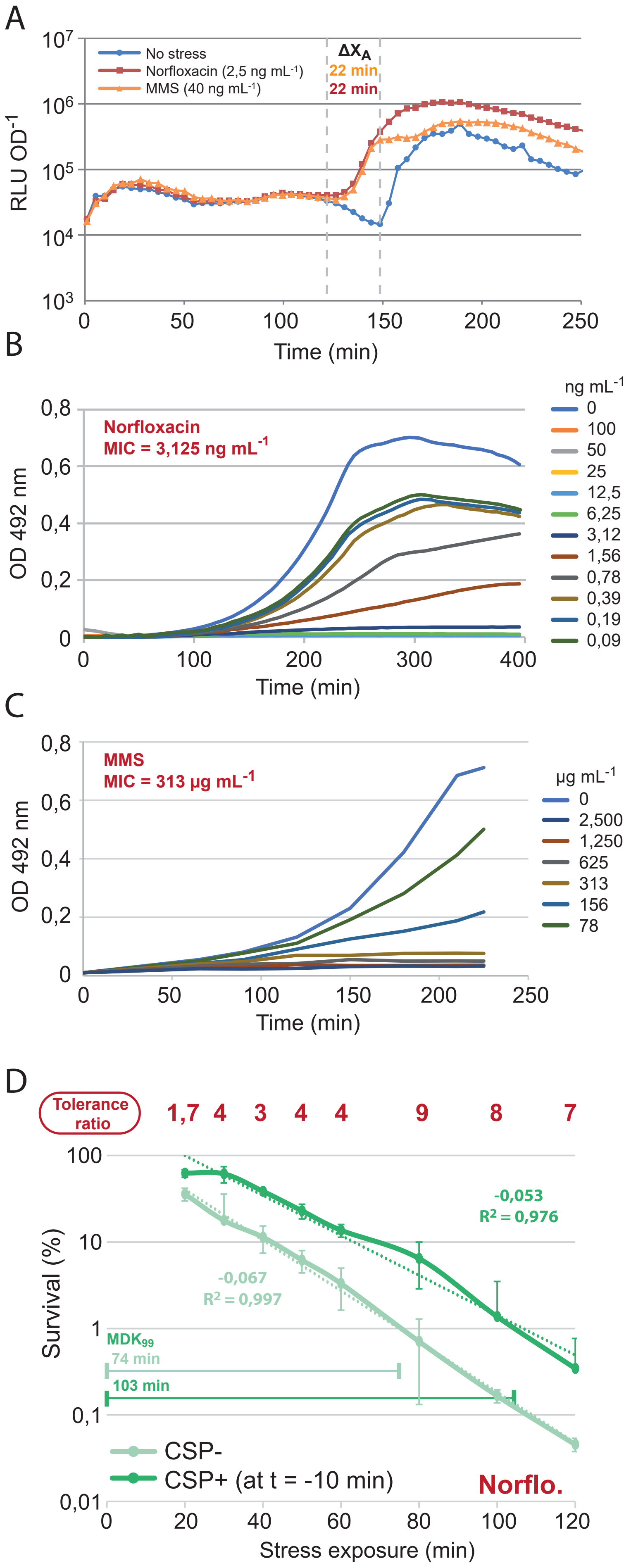
A
No stress
Norfloxacin (2,5 ng mL-1)
MMS (40 ng mL-1)
ΔXA
22 min
22 min
RLU OD-1
Time (min)
B
Norfloxacin
MIC = 3,125 ng mL-1
OD 492 nm
ng mL-1
0
100
50
25
12,5
6,25
3,12
1,56
0,78
0,39
0,19
0,09
Time (min)
C
MMS
MIC = 313 µg mL-1
OD 492 nm
µg mL-1
0
2,500
1,250
625
313
156
78
Time (min)
D
Tolerance ratio
1,7 4 3 4 4 9 8 7
-0,053
R2 = 0,976
-0,067
R2 = 0,997
MDK99
74 min
103 min
Survival (%)
CSP-
CSP+ (at t = -10 min)
Norflo.
Stress exposure (min)

Supplementary Figure 10: MIC calculation, competence induction and survival time course of Norflo and MMS. (A) Exposure to Norflo or MMS at sub-MIC levels reduces the X_A period of competence in R895 *comC*$^+$ cells able to spontaneously develop competence. Competence was visualised using P_{ssbB}::*luc*. Stresses added at 0 min. Data representative of triplicate repeats. (B) Growth of pneumococci in a gradient of norfloxacin concentrations to determine MIC, defined as the lowest concentration blocking pneumococcal growth. Data representative of triplicate repeats. (C) Growth of pneumococci in a gradient of MMS concentrations to determine MIC, defined as in panel B. Data representative of triplicate repeats. (D) Time-course of survival of competent (dark green) and non-competent (light green) R1501 cells exposed to norfloxacin. Tolerance ratios calculated as in Figure 3A. Dotted lines represent exponential fits with exponential rates and R^2 values provided. MDK_{99} values represent time taken to kill 99 % of the population. Means and standard deviations calculated from triplicate repeats. (C) Luminometry measurements used for calculation of X_A times in Figure 2CD. The R4428 strain was grown in non-permissive medium and separated into two samples. The first sample was exposed to CSP during 1 minute at 37°C and the other not (Figure 2C and Methods). Competence (specific activity readings in RLU OD^{-1}) is reported every minute. The thick dark green curve corresponds to the assay with only cells exposed to CSP (Activated) and thick light green curve to the assay with only cells not exposed to CSP (Non-activated). The other coloured curves correspond respectively to mixes of Activated/Non-activated cells as depicted in the left graph. Right graph corresponds to the mix without activated cell using the same colour guide as control. Means and standard deviations are reported from triplicate repeats.

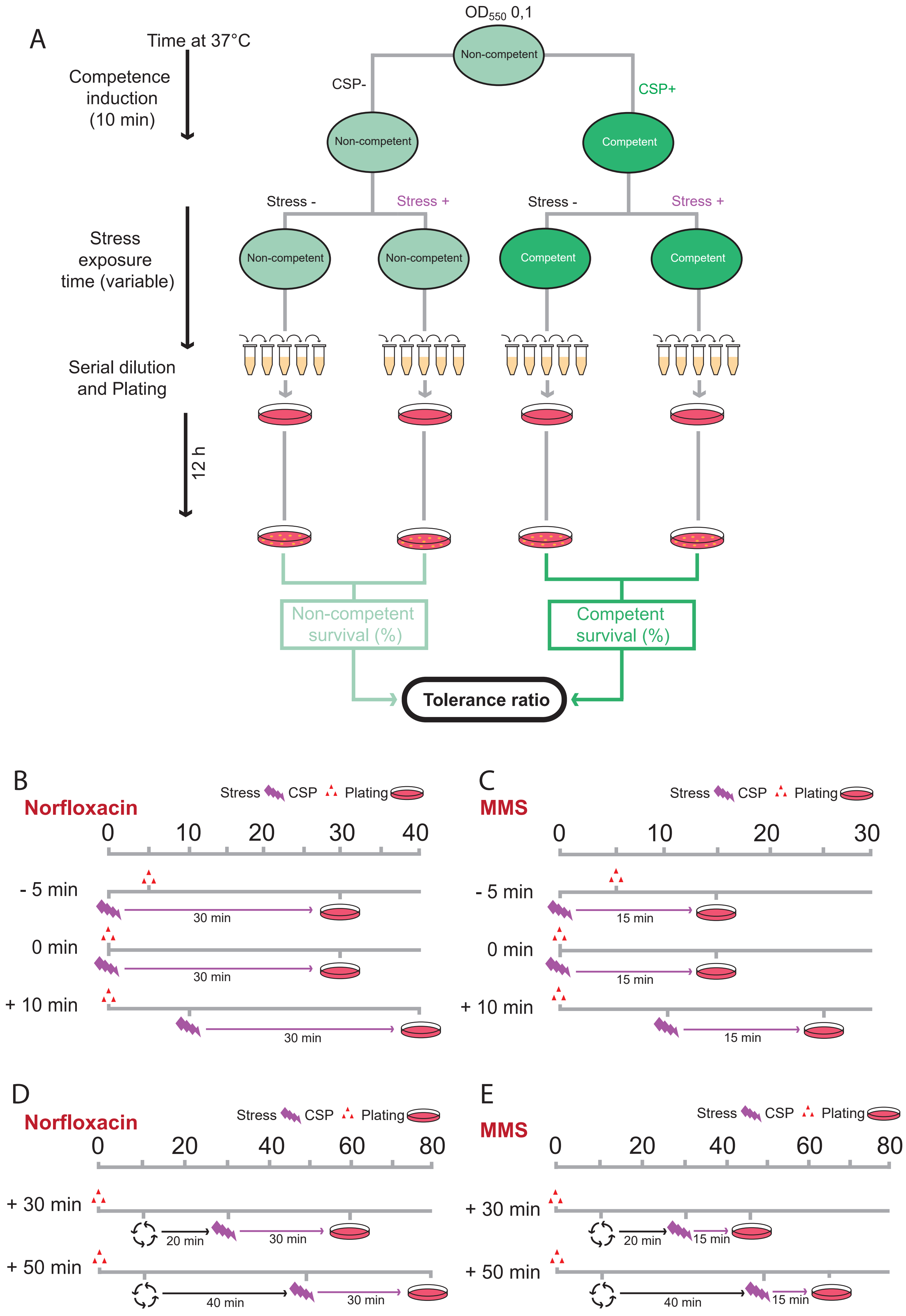
A
Time at 37°C
Competence induction (10 min)
Stress exposure time (variable)
Serial dilution and Plating
12 h
OD550 0,1
Non-competent
CSP-
CSP+
Non-competent
Competent
Stress -
Stress +
Stress -
Stress +
Non-competent
Non-competent
Competent
Competent
Non-competent survival (%)
Competent survival (%)
Tolerance ratio
B
Norfloxacin
Stress CSP Plating
0 10 20 30 40
- 5 min
30 min
0 min
30 min
+ 10 min
30 min
C
MMS
Stress CSP Plating
0 10 20 30
- 5 min
15 min
0 min
15 min
+ 10 min
15 min
D
Norfloxacin
Stress CSP Plating
0 20 40 60 80
+ 30 min
20 min
30 min
+ 50 min
40 min
30 min
E
MMS
Stress CSP Plating
0 20 40 60 80
+ 30 min
20 min
15 min
+ 50 min
40 min
15 min

Supplementary Figure 11: Schematic representations of survival assays. (A) Pre-competent cells were grown to OD_{550} 0,1 and split into two cultures, with one half induced to competence by addition of 100 ng mL^{-1} synthetic CSP. After 10 min at 37 °C, cultures were again split and exogenous stress was added or not, before further incubation at 37 °C for variable time. Cells were then serially diluted and plated before a final incubation at 37 °C for 12 h. Comparing cfu from stressed and non-stressed conditions allowed calculation of survival ratios for competent and non-competent cells, and comparison of these ratios produced the tolerance ratio, revealing the effect of competence on tolerance to a particular stress. (B) Schematic representation of timings of Norflo survival assays exploring exposure to stress at different times relative to CSP addition. (C) Schematic representation of timings of MMS survival assays exploring exposure to stress at different times relative to CSP addition. (D) Schematic representation of timings of Norflo survival assays exploring exposure to stress at time points after CSP addition. (E) Schematic representation of timings of MMS survival assays exploring exposure to stress at time points after CSP addition.

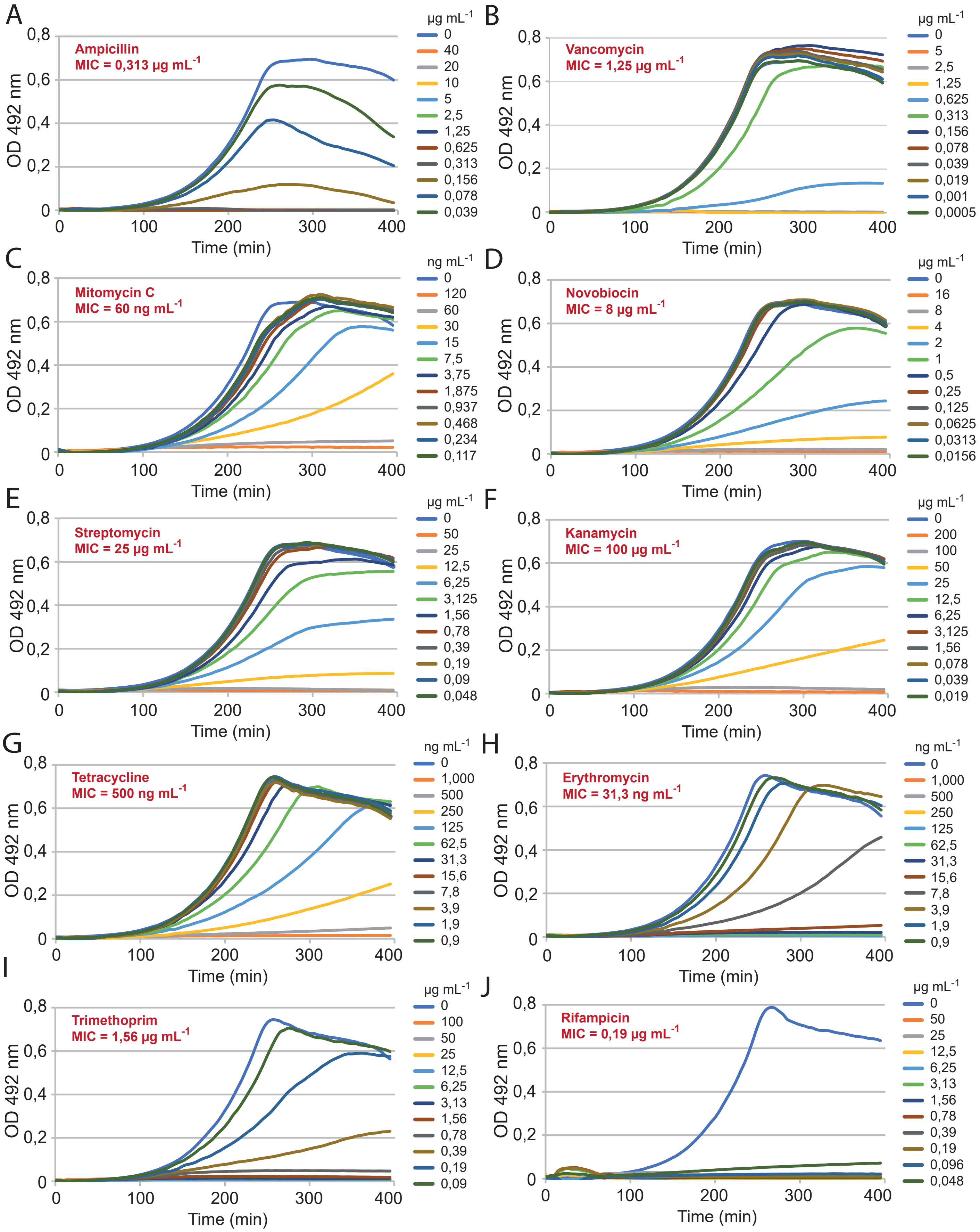
A
Ampicillin
MIC = 0,313 µg mL-1
OD 492 nm
Time (min)
µg mL-1
0
40
20
10
5
2,5
1,25
0,625
0,313
0,156
0,078
0,039
B
Vancomycin
MIC = 1,25 µg mL-1
µg mL-1
0
5
2,5
1,25
0,625
0,313
0,156
0,078
0,039
0,019
0,001
0,0005
C
Mitomycin C
MIC = 60 ng mL-1
ng mL-1
0
120
60
30
15
7,5
3,75
1,875
0,937
0,468
0,234
0,117
D
Novobiocin
MIC = 8 µg mL-1
µg mL-1
0
16
8
4
2
1
0,5
0,25
0,125
0,0625
0,0313
0,0156
E
Streptomycin
MIC = 25 µg mL-1
µg mL-1
0
50
25
12,5
6,25
3,125
1,56
0,78
0,39
0,19
0,09
0,048
F
Kanamycin
MIC = 100 µg mL-1
µg mL-1
0
200
100
50
25
12,5
6,25
3,125
1,56
0,078
0,039
0,019
G
Tetracycline
MIC = 500 ng mL-1
ng mL-1
0
1,000
500
250
125
62,5
31,3
15,6
7,8
3,9
1,9
0,9
H
Erythromycin
MIC = 31,3 ng mL-1
ng mL-1
0
1,000
500
250
125
62,5
31,3
15,6
7,8
3,9
1,9
0,9
I
Trimethoprim
MIC = 1,56 µg mL-1
µg mL-1
0
100
50
25
12,5
6,25
3,13
1,56
0,78
0,39
0,19
0,09
J
Rifampicin
MIC = 0,19 µg mL-1
µg mL-1
0
50
25
12,5
6,25
3,13
1,56
0,78
0,39
0,19
0,096
0,048

Figure S12

Supplementary Figure 12: Growth curves for calculation of MIC of tested stresses. Pneumococci in a gradient of stress concentrations to determine MIC, defined as in Supplementary Figure 10. Data representative of triplicate repeats. (A) Ampicillin. (B) Vancomycin. (C) MMC. (D) Novobiocin. (E) Streptomycin. (F) Kanamycin. (G) Tetracycline. (H) Erythromycin. (I) Trimethoprim. (J) Rifampicin.

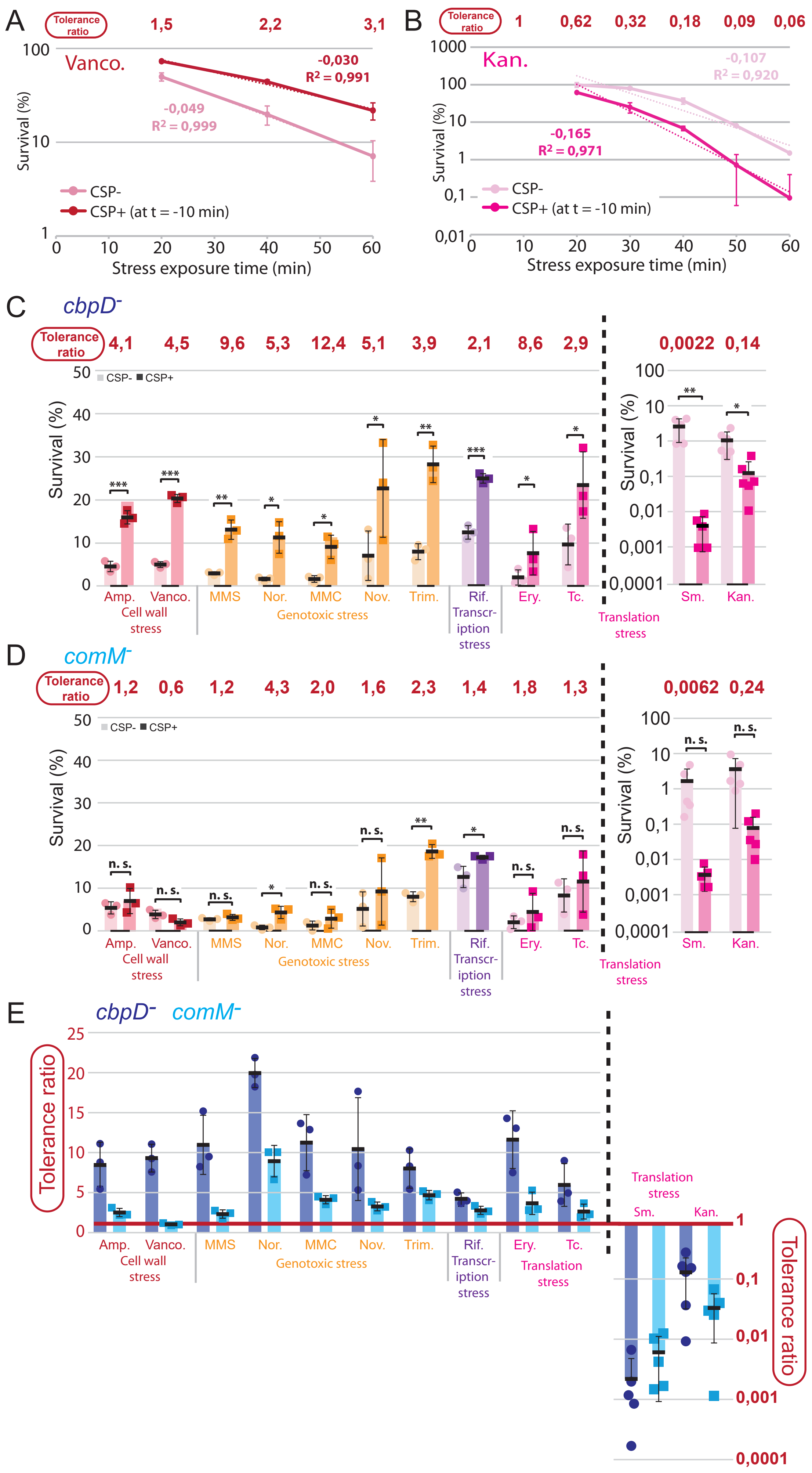
A
Tolerance ratio
1,5
2,2
3,1
Vanco.
-0,030
R² = 0,991
-0,049
R² = 0,999
Survival (%)
CSP-
CSP+ (at t = -10 min)
Stress exposure time (min)
B
Tolerance ratio
1
0,62
0,32
0,18
0,09
0,06
Kan.
-0,107
R² = 0,920
-0,165
R² = 0,971
Survival (%)
CSP-
CSP+ (at t = -10 min)
Stress exposure time (min)
C
cbpD-
Tolerance ratio
4,1 4,5 9,6 5,3 12,4 5,1 3,9 2,1 8,6 2,9
0,0022 0,14
CSP- CSP+
Survival (%)
Amp. Vanco. MMS Nor. MMC Nov. Trim. Rif. Ery. Tc.
Cell wall stress
Genotoxic stress
Transcr-iption stress
Translation stress
Sm. Kan.
D
comM-
Tolerance ratio
1,2 0,6 1,2 4,3 2,0 1,6 2,3 1,4 1,8 1,3
0,0062 0,24
CSP- CSP+
Survival (%)
n. s.
Amp. Vanco. MMS Nor. MMC Nov. Trim. Rif. Ery. Tc.
Cell wall stress
Genotoxic stress
Transcr-iption stress
Translation stress
Sm. Kan.
E
cbpD- comM-
Tolerance ratio
Translation stress
Sm. Kan.
Amp. Vanco. MMS Nor. MMC Nov. Trim. Rif. Ery. Tc.
Cell wall stress
Genotoxic stress
Transcr-iption stress
Translation stress
Tolerance ratio

Supplementary Figure 13: Survival assay control experiments in wildtype, *cbpD*$^-$ and *comM*$^-$ cells. (A) Time-course of survival of competent and non-competent R1501 cells exposed to vancomycin. Tolerance ratios calculated as in Figure 3A. Dotted lines represent exponential fits with exponential rates and R^2 values provided. (B) Time-course of survival of competent and non-competent R1501 cells exposed to kanamycin. Tolerance ratios calculated as in Figure 3A. Dotted lines represent exponential fits with exponential rates and R^2 values provided. (C) Survival of competent and non-competent *cbpD*$^-$ cells (R4951) exposed to various stresses for 60 min starting at +10 min relative to CSP addition. Experimental procedures and representations as in Figure 4A. p values, Amp, 0,0005; Vanco, <0,0001; MMS, 0,0015; Nor, 0,0105; MMC, 0,0104; Nov, 0,0485; Trim, 0,0016; Rif, 0,0004; Ery, 0,046; Tc, 0,041; Sm, 0,0085; Kan, 0,039. *, p < 0,05; **, p < 0,01; ***, p < 0,001. (D) Survival of competent (dark) and non-competent (light) *comM*$^-$ cells (R4590) exposed to various stresses for 60 min starting at +10 min relative to CSP addition. Experimental procedures and representations as in Figure 4A. p values, Amp, 0,4434; Vanco, 0,0653; MMS, 0,369; Nor, 0,0161; MMC, 0,319; Nov, 0,468; Trim, 0,008; Rif, 0,0353; Ery, 0,418; Tc, 0,527; Sm, 0,0993; Kan, 0,0559. n.s., non-significant, p > 0,05; *, p < 0,05. (E) Comparison of tolerance ratios of *cbpD*$^-$ and *comM*$^-$ cells, with values as in Figure 4C.

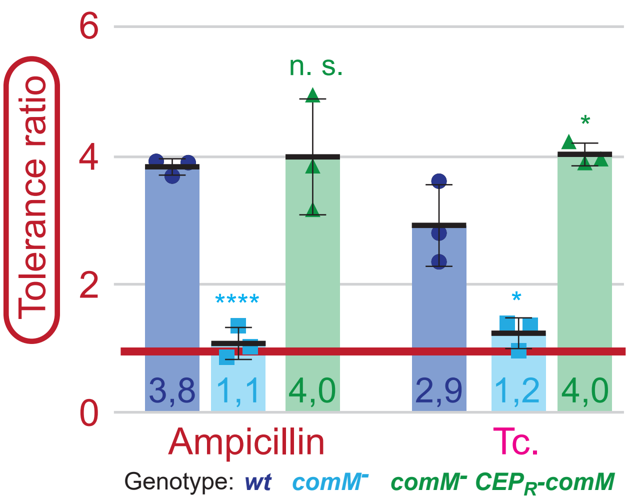

6
4
2
0
Tolerance ratio
n. s.
****
*
*
3,8
1,1
4,0
2,9
1,2
4,0
Ampicillin
Tc.
Genotype: wt comM- comM- CEPR-comM

Supplementary Figure 14: Ectopic expression of *comM* under P_R transcriptional control restores tolerance increase in competent *comM*⁻ mutant cells. Comparison of tolerance ratios of wildtype, *comM*$^-$ and *comM*$^-$, *CEP$_R$-comM* strains exposed to ampicillin or tetracycline for 60 min starting at +10 min relative to CSP addition. Experimental procedures as in Figure 4A, representations as in Figure 4C. Ectopic expression of *comM* was by addition of BIP peptide (250 ng μL^{-1}) concurrently with CSP. p values, Amp *comM*$^-$, <0,0001; Amp *comM*$^-$ *CEP$_R$-comM*, 0,781; MMC *comM*$^-$, 0,0129; MMC *comM*$^-$ *CEP$_R$-comM*, 0,044. n.s., non-significant, p > 0,05; *, p < 0,05; ****, p < 0,001.

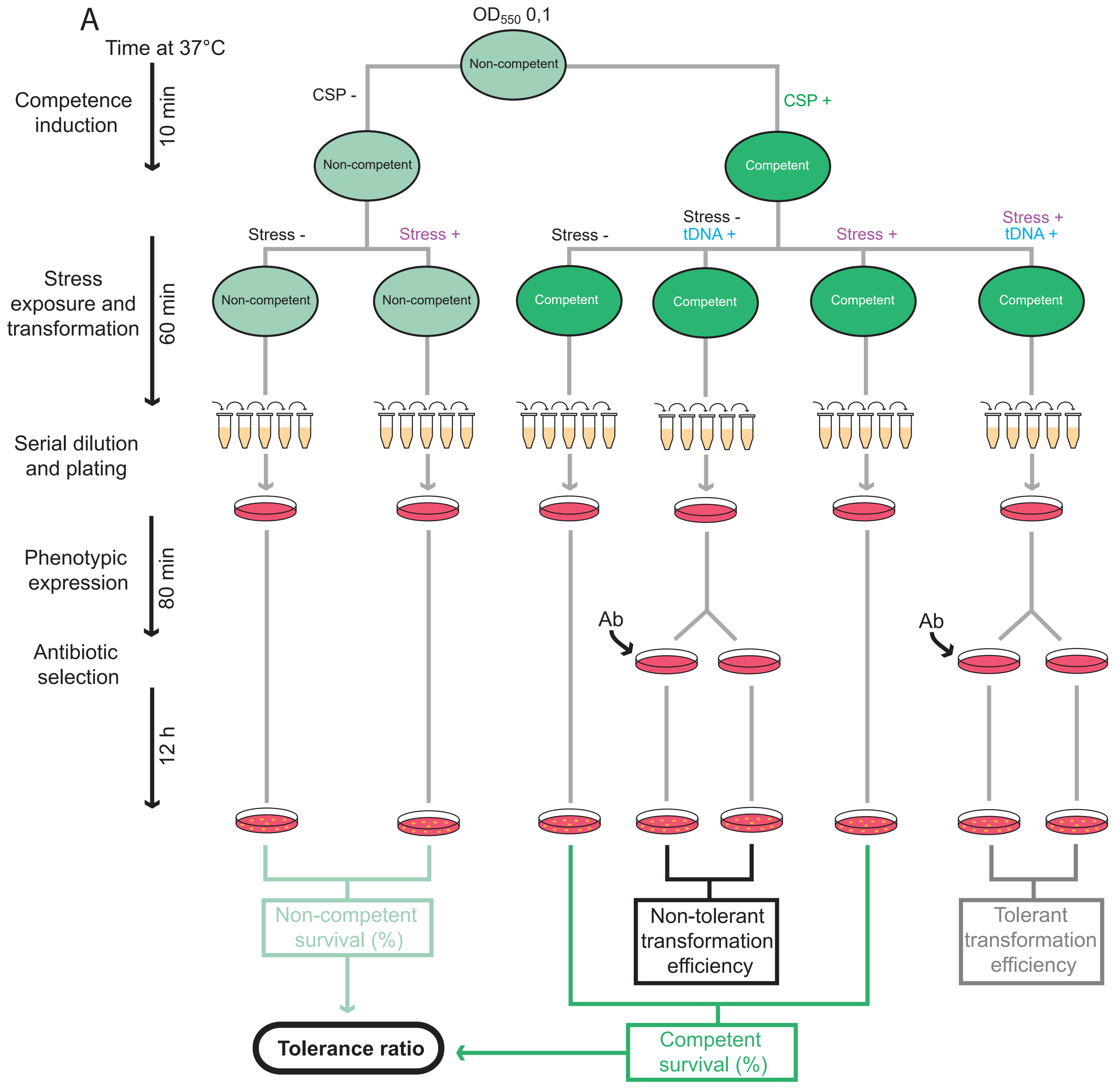
A
Time at 37°C
Competence induction
10 min
Stress exposure and transformation
60 min
Serial dilution and plating
Phenotypic expression
80 min
Antibiotic selection
12 h
OD550 0,1
Non-competent
CSP -
CSP +
Non-competent
Competent
Stress -
Stress +
Stress -
Stress -
tDNA +
Stress +
Stress +
tDNA +
Non-competent
Non-competent
Competent
Competent
Competent
Competent
Ab
Ab
Non-competent survival (%)
Non-tolerant transformation efficiency
Tolerant transformation efficiency
Tolerance ratio
Competent survival (%)

Supplementary Figure 15: Schematic representation of transformation assay. (A) Pre-competent R3369 cells were grown to OD_{550} 0,1 and split into two cultures, with one half induced to competence by addition of synthetic CSP. After 10 min at 37°C, cultures were again split and tDNA and exogenous stress were added as shown, before a further 60 min incubation at 37°C. Cells were then serially diluted and plated before 80 min of phenotypic expression at 37°C. A second layer of medium with selective antibiotic was added to desired $tDNA^+$ plates to select for transformants. Comparison of cfu in stress +/- conditions in CSP+ or CSP- cells determined the tolerance of competent and non-competent cells in the face of the stress. Comparing these values revealed the effect of competence on stress tolerance via the tolerance ratio. Transformation efficiencies of stress +/- populations were determined by comparing cfu on selective and non-selective plates.

References

1. Prudhomme, M., Berge, M., Martin, B. & Polard, P. Pneumococcal Competence Coordination Relies on a Cell-Contact Sensing Mechanism. *PLoS Genet.* **12**, e1006113 (2016).
2. Moreno-Gámez, S. *et al.* Quorum sensing integrates environmental cues, cell density and cell history to control bacterial competence. *Nat Commun* **8**, 854 (2017).
3. Prudhomme, M.; Claverys, J-P. There will be a light: the use of luc transcriptional fusions in living pneumococcal cells. in *The Molecular Biology of Streptococci.* 519–524 (Hakenbeck, R.; Chatwal, GS, 2007).
4. Bergé, M. J. *et al.* Midcell recruitment of the DNA uptake and virulence nuclease, EndA, for pneumococcal transformation. *PLoS Pathog.* **9**, e1003596 (2013).
5. Hotchkiss, R. D. CYCLICAL BEHAVIOR IN PNEUMOCOCCAL GROWTH AND TRANSFORMABILITY OCCASIONED BY ENVIRONMENTAL CHANGES. *Proc Natl Acad Sci U S A* **40**, 49–55 (1954).
6. Suzuki, H., Pangborn, J. & Kilgore, W. W. Filamentous cells of Escherichia coli formed in the presence of mitomycin. *J. Bacteriol.* **93**, 683–688 (1967).
7. Mortier-Barrière, I., de Saizieu, A., Claverys, J. P. & Martin, B. Competence-specific induction of recA is required for full recombination proficiency during transformation in Streptococcus pneumoniae. *Mol. Microbiol.* **27**, 159–170 (1998).
8. Chastanet, A., Prudhomme, M., Claverys, J. P. & Msadek, T. Regulation of Streptococcus pneumoniae clp genes and their role in competence development and stress survival. *J. Bacteriol.* **183**, 7295–7307 (2001).
9. Kurushima, J. *et al.* Unbiased homeologous recombination during pneumococcal transformation allows for multiple chromosomal integration events. *Elife* **9**, e58771 (2020).

10. Bergé, M., Moscoso, M., Prudhomme, M., Martin, B. & Claverys, J.-P. Uptake of transforming DNA in Gram-positive bacteria: a view from Streptococcus pneumoniae. *Mol. Microbiol.* **45**, 411–421 (2002).

Supplementary Table 1 - Competence induction, plating and minimum inhibitory concentration (MIC) details for stresses used.

				Figure 10 and Supplementary Figure 12	Figures 3-5 and Supplementary Figures 10-15
**Stress**	**Plating concentration (µg mL^{-1})**	**Non-inducing stress range (µg mL^{-1})**	**Inducing stress range (µg mL^{-1})**	**MIC (µg mL^{-1})**	**Survival assay 60 min (µg mL^{-1})**
Ampicillin	nd	0,008 - 1 *		3,125	40
Vancomycin	nd	0,039 - 5 *		1,25	5
MMS	nd		40	313	625
Norfloxacin	nd		5 - 15 *	3,125	100
Mitomycin C	nd		0,04 - 0,06 *	0,06	0,12
Novobiocin	4	0,03 - 8 *		8	16
Streptomycin	100		6,12 - 25 *	25	50
Kanamycin	250		31 -125 *	100	200
Tetracyclin	1	0,0039 - 0,5 *		0,5	1
Erythromycin	0,2	0,0039 - 0,5 *		0,031	1
Rifampicin	2	0,0024 - 0,31 *		0,09	50
Trimethoprim	20		0,7 **	1,56	100

* [33]

** [34]

nd, not determined

Supplementary Table 2 - Survival and tolerance ratios from transformation assays

Strain	Genotype	Stress	Donor DNA	Figure reference	Tolerance ratio
R3369	*comC2D1*	Vancomycin	*comFA::kan*	Figure 5B	**3**
R3369	*comC2D1*	Trimethoprim	*comFA::kan*	Figure 5B	**3**
R3369	*comC2D1*	Erythromycin	*comFA::kan*	Figure 5B	**4**
R3369	*comC2D1*	Norfloxacin	*comFA::kan*	Figure 5B	**6**
R3369	*comC2D1*	Ampicillin	*comFA::kan*	Figure 5B	**3**
R3369	*comC2D1*	Vancomycin	*rpsL41*	Figure 5C	**6**
R3369	*comC2D1*	Trimethoprim	*rpsL41*	Figure 5C	**3**
R3369	*comC2D1*	Erythromycin	*rpsL41*	Figure 5C	**3**
R3369	*comC2D1*	Norfloxacin	*rpsL41*	Figure 5C	**7**
R3369	*comC2D1*	Ampicillin	*rpsL41*	Figure 5C	**4**
R3369	*comC2D1*	Vancomycin	*rpoB*	Figure 5D	**4**
R3369	*comC2D1*	Trimethoprim	*rpoB*	Figure 5D	**4**
R3369	*comC2D1*	Erythromycin	*rpoB*	Figure 5D	**4**
R3369	*comC2D1*	Norfloxacin	*rpoB*	Figure 5D	**6**
R3369	*comC2D1*	Ampicillin	*rpoB*	Figure 5D	**3**
R3369	*comC2D1*	Vancomycin	*rpsL41*	Figure 5E	**6**
R3369	*comC2D1*	Trimethoprim	*rpsL41*	Figure 5E	**5**
R3369	*comC2D1*	Vancomycin	*rpoB*	Figure 5E	**4**
R3369	*comC2D1*	Trimethoprim	*rpoB*	Figure 5E	**4**
R4711	*comC2D1, hexA::ermAM*	Vancomycin	*rpsL41*	Figure 5E	**7**
R4711	*comC2D1, hexA::ermAM*	Trimethoprim	*rpsL41*	Figure 5E	**6**
R4711	*comC2D1, hexA::ermAM*	Vancomycin	*rpoB*	Figure 5E	**4**
R4711	*comC2D1, hexA::ermAM*	Trimethoprim	*rpoB*	Figure 5E	**5**

Supplementary Table 3: Strains, plasmids and primers used in this study

Strain	Genotype; phenotype	Source/reference
D39	Cps$^+$ (serotype II)	NCTC 7466
D39$_V$	Cps+ (serotype II), D39 from Veening laboratory, NCTC 7466 derivative	[78]
R304	R800 but *nov1, rif23, rpsL41* ; Nov^R, Rif^R, Sm^R	[80]
R895	R800 but *ssbB* :: pR424(*luc*); *ssbB* $^+$; Cm^R, Cps$^-$	[81]
R1501	R800 but *comC0*	[11]
R1620	*rpsL1, cbpD::spc* ^{3C}; Sm^R, Spc^R	[77]
R2287	R1501 but *comFA::kan* ^{3C}; Kan^R	This study
R2737	R895 but CEP^M; Cm^R, Kan^R	[76]
R3316	*ssbB::pR424(luc),* ^{e} *P* $_{lac}$ *::dnaC* ; *ssbB+* , Cm^R, Spc^R	[36]
R3369	R800 but *comC* $_2$ *D* $_1$	[18]
R3967	*comC0, cbpD* ^{C75A} *, comM::cat* ; Cm^R	[15]
R4428	R2737 but Δ7pb at +77 pb from the kanamycin start codon open reading frame; Cm^R, Kan^s	This study
R4590	R3369 but *comM::cat* ; Cm^R	This study
R4591	R3369 but *cbpD::spc* ; Spc^R	This study
R4592	R4590 but *cbpD::spc* ; Cm^R , Spc^R	This study
R5218	R4590 but *CEP* $_R$ *-comM* ; Cm^R, Kan^R	This study
TCP1253	*hexB* $_{Rx}$ *;mal ;rpsL* $_1$ *;bgl* $_1$ *;ssbB* ::*pR424(luc,Cm); comA* ::*Kan* ^{42}; Hex$^-$, Mal$^-$, Sm^R, Cm^R, Km^R, Cps$^-$	[57]
TD82	D39 *ssbB* ::*pR424(luc)* ; *ssbB* $^+$; Cm^R, Cps$^-$	[39]
TD277	D39 *ssbB* ::*pR424(luc)* ; *ssbB* $^+$; Cm^R, Cps$^+$ (serotype II)	This study
TD288	D39$_V$ *ssbB* ::*pR424(luc)* ; *ssbB* $^+$; Cm^R, Cps$^+$ (serotype II)	This study
84	TD288 but *endA::kan* ; *ssbB* $^+$; Cm^R, Cps$^+$ (serotype II), Kan^R	[82]
DLA3	D39$_V$, Δ*bgaA* ::(P$_{ssbB}$ ::*luc* , tet ^{R}) ; Tet^R, Cps$^+$ (serotype II)	[34]

Plasmid	Genotype	Source/reference
pR410	pEMcat derivative, Amp^R, Kan^R; carries a Kan^R mariner mini-transposon (1337 bp)	[55]

Primer	Sequence (5'-3')	Source/reference	Use
CJ339	CTTGTTCAGCTTTTTCAATCATGCT	This study	Amplification of *comFA::kan* PCR fragment
CJ356	ATACAAAACACCGGAAGAAGCATCC	This study	Amplification of *comFA::kan* PCR fragment
comFA3	AAATTCAATTTCATAACACC	[83]	Mariner mutagenesis of *comFA*
comFA4	ACGATGTTGTTAAGTTTAAC	[83]	Mariner mutagenesis of *comFA*
MB117	AATCTCCGCTGTAGGTCACTTTCTT	[78]	Amplification of *rpsL41* PCR fragment
MB120	TTGGATTGGGTGTGCATTTGC	[78]	Amplification of *rpsL41* PCR fragment
MB137	CGTCTAGGACACGCATGTCAAGA	[78]	Amplification of *rpoB* PCR fragment
MB138	GGCGGTAGACGGATTTGAACC	[78]	Amplification of *rpoB* PCR fragment
MP259	CCTTTCTTTTTTGATGTTCA	This study	Generation of Kan^S 7 bp deletion allele - 5' fragment
MP260	ACCAGCTTATATACCTTAGCATTCCTTCCGTATCTTTCAC	This study	Generation of Kan^S 7 bp deletion allele - 5' fragment
MP261	GTGAAAGATACGGAAGGAATGCTAAGGTATATAAGCTGGT	This study	Generation of Kan^S 7 bp deletion allele - 3' fragment
MP262	TGTTGCTGTCTCCCAGGTCG	This study	Generation of Kan^S 7 bp deletion allele - 3' fragment
OCN75	Cy3-AATTGGTTCGCAAACCGCGTA	[41]	Amplification of Cy3-labelled fluorescent DNA
OCN76	Cy3-TTACACGTCCACCGCGAAGAA	[41]	Amplification of Cy3-labelled fluorescent DNA
